# Efficacy of Parent-Infant-Psychotherapy with mothers with postpartum mental disorder: study protocol of the randomized controlled trial as part of the SKKIPPI project

**DOI:** 10.1186/s13063-020-04443-7

**Published:** 2020-06-05

**Authors:** J. Mattheß, M. Eckert, K. Richter, G. Koch, T. Reinhold, P. Vienhues, A. Berghöfer, S. Roll, T. Keil, F. Schlensog-Schuster, K. von Klitzing, C. Ludwig-Körner, L. Kuchinke

**Affiliations:** 1 International Psychoanalytic University, Stromstr. 3b, 10555 Berlin, Germany; 2 Department of Psychiatry, Psychosomatics and Psychotherapy, Diakonissenkrankenhaus Flensburg, Flensburg, Germany; 3 Institute for Social Medicine, Epidemiology and Health Economics, Charité – Universitätsmedizin, Berlin, Germany; 4 Institute of Clinical Epidemiology and Biometry, University of Wuerzburg, Wuerzburg, Germany; 5 State Institute of Health, Bavarian Health and Food Safety Authority, Bad Kissingen, Germany; 6 Department of Child and Adolescent Psychiatry, Psychotherapy and Psychosomatics, University of Leipzig, Leipzig, Germany

**Keywords:** Randomized controlled trial, Parent-infant-psychotherapy, Attachment, Maternal sensitivity, Postpartum mental disorder, Inpatient psychiatric department, Home visits

## Abstract

**Background:**

After the birth of a child, many mothers and fathers experience postpartum mental disorders like depression, anxiety, obsessive-compulsive disorder, stress or other illnesses. This endangers the establishment of a secure attachment between the children and their primary caregivers. Early problems in parent-child interaction can have adverse long-term effects on the family and the child’s well-being. In order to prevent a transgenerational transmission of mental disorders, it is necessary to evaluate psychotherapeutic interventions that target psychologically burdened parents of infants or toddlers. The aim of this trial is to investigate the efficacy of Parent-Infant-Psychotherapy (PIP) for mothers with postpartum mental disorder and their infants (0–12 months).

**Methods/design:**

In this open, randomized controlled intervention trial 180 mother-infant-dyads will be included and randomly allocated to 12 sessions of PIP or care as usual. The interventions take place either in inpatient adult psychiatric departments or in outpatient settings with home visits. The primary outcome is the change in maternal sensitivity assessed by the Sensitivity subscale of the Emotional Availability Scale (EAS) through videotaped dyadic play-interactions after 6 weeks. Secondary outcomes are maternal psychopathology, stress, parental reflective functioning, infant development and attachment after 6 weeks and 12 months. In addition, maternal attachment (AAI) and reflective functioning (AAI) will be analyzed as potential moderators, and resource usage in the German health system as well as associated costs will be evaluated.

**Discussion:**

There is increasing demand for well-controlled studies on psychotherapeutic interventions in the postpartum period that do not only focus on particular risk groups. This randomized controlled trial (RCT) represents one of the first studies to investigate the efficacy of PIP in inpatient psychiatric departments and outpatient care centers in Germany. The results will fill knowledge gaps on the factors contributing to symptom reduction in postpartum mental disorders and improvements in mother-child relationships and help in developing preventive and therapeutic strategies for the fragmented German health care system.

**Trial registration:**

German Register for Clinical Trials, ID: DRKS00016353.

## Background

Parental mental health problems during infancy are known to impact children’s emotional well-being and to modulate future developmental trajectories [1–3]. Postpartum mental health problems show high prevalence rates. In the first year after the birth of a child up to 22% of all mothers develop a postpartum depression (PPD) [4], and nearly 21% of all women suffer from postpartum anxiety [1] or obsessive-compulsive illnesses [5] with a high comorbidity of these disorders [6]. Parental mental health problems have been identified as major factors for problems in parent-child interaction and as risk factors for the child’s well-being [7, 8]. In contrast, a positive mother-child relationship is an essential resilience factor for child development. Maternal sensitivity towards the infant is seen as a key variable in mother-child interaction and secure attachment development [9–11]. The lack of sensitivity and the low level of parental skills associated with PPD are known to affect the interaction with the child which has adverse effects on the child’s development [12]. According to Lyons-Ruth [2], these malignant patterns increase the risk of disorganized attachment patterns and produce child psychopathologies [13]. Recent reviews emphasize that this relationship might be more complex and dependent on the severity of the problems and the presence of additional risk factors [14]. The available evidence thus favors interventions that focus on altering maternal or paternal sensitivity which in turn are more effective in enhancing attachment security [1, 4, 14].

Parent-Infant-Psychotherapy (PIP) has been developed as an intervention that aims to improve the attachment relationship between an infant and their parents to pave the way for a healthy child development [1, 15, 16]. PIP aims not only to reduce parental postpartum psychopathological symptoms but also to establish stable relationship patterns and to improve maternal or paternal sensitivity [15, 17, 18]. In comparison to other interventions, such as parenting programs, family support and medication [19–21], PIP shows high effect sizes on attachment development [15, 22]. Barlow et al. (see [15, 22]) recently reviewed eight clinical trials comprising either comparisons of PIP with no-treatment controls or other types of control groups. There was no difference in the efficacy of improving maternal sensitivity between all types of interventions, but PIP was significantly more effective in improving attachment security. While the meta-analysis of Barlow et al. did not reveal differences for measures of parental sensitivity [1], the authors clearly noted that the quality of the included studies was low while heterogeneity of these samples was high. The available evidence seems to indicate that PIP is more effective in subgroups of at-risk populations but more research addressing these limitations is needed [1, 23, 24].

This seems to be particularly true in case of mothers with mental health problems and young infants. Only four randomized controlled trials (RCTs) examining PIP with infants under 1 year of age have been published [1, 25–27]. These studies clearly differed in the addressed subpopulations (imprisoned mothers [25], depressed mothers [28], mothers experiencing social adversity [1], diverse maternal concerns and problems [26]) and the investigated settings (prison [25], outpatient setting [26], clinical setting [1] and home visits [28]). Only the RCT of Cooper et al. [28] required that study participants have a diagnosis of PPD as an inclusion criteria, while in the other studies participants had not to fulfill specific diagnoses at the time of recruitment [25, 26] or only research-related cut-offs for mental health problems [1]. Only one of these four studies [26] found evidence for an improvement of maternal sensitivity at post-intervention measurements and none revealed effects on infants’ attachment. Thus, there is currently neither evidence for the effectiveness of PIP with infants under 1 year of age nor conclusive evidence regarding clinical settings and psychiatric diagnoses. It therefore remains unclear whether this is due to the subpopulations examined, or the diagnostic criteria applied or the setting.

Thus, despite the clear benefits of attachment- and sensitivity-related interventions for the mother-child-dyad [19], a corresponding study of PIP with validation in inpatient psychiatric departments is still needed. Moreover, recent developments in child psychiatry provide evidence for the effectiveness of home treatment or home-based interventions [29–31]. Home visits by psychotherapists have the advantage of an intervention in the patient’s own environments, which might be particularly helpful for mothers in the postpartum period. It is assumed that staying in one’s own environment, and with the familiar daily routine, may have a positive influence on the treatment outcome. PIP home-based interventions have demonstrated to effectively improve the children’s attachment organization [32], but null effects on attachment measures and maternal sensitivity have also been reported [33, 34]. Again, there are large methodological differences between these studies.

In summary, previous randomized trials investigated the positive effect of PIP on the attachment quality of infants (and long-term effects up to preschool age [35]). In contrast to the present study, most of these studies have focused exclusively on specific risk groups or have taken place in outpatient settings and home visits but with relatively low diagnostic cut-offs [1, 25, 26, 28, 34]. The efficacy of PIP in inpatient departments and home-based interventions for mothers with diagnosed postpartum mental disorder will be investigated in this randomized controlled efficacy study.

## Methods/design

### Overview

The Parent-Infant-Psychotherapy in cohort- and intervention studies [Eltern-Säugling-KleinKInd-Psychotherapie mittels Prävalenz- und Interventionsstudien] (SKKIPPI) research project (www.skkippi.de) is a multicenter research project in Germany located in Berlin, Flensburg, Hamburg and Leipzig with the aim to examine epidemiology and treatment of psychosocial and mental disorders in the postpartum period. SKKIPPI is divided into an epidemiological cohort study that evaluates the prevalence of psychosocial and mental disorders in the postpartum period, the utilization of health and social care services, and their economic burden, and two randomized controlled intervention studies to evaluate the efficacy of PIP.

### Aims and hypotheses

The present RCT will be designed to evaluate the efficacy of focused PIP interventions in inpatient adult psychiatric departments with Mother and Baby Units (MBUs) or in outpatient psychiatric departments or via home visits. The decision about setting (inpatient or outpatient) will depend on the severity of the symptoms and/or maternal consent. It is hypothesized that in comparison to routine therapy (care as usual, CAU) mothers with mental disorders and their infants under 1 year of age will benefit from an increase in maternal sensitivity after 6 weeks of intervention. It will be expected that the routine therapy also reduces the psychopathological symptoms, but nevertheless has little or no effect on maternal sensitivity.

#### Secondary hypotheses

1. In comparison to routine therapy, PIP will be effective in reducing maternal psychopathological symptoms and psychological distress for mothers with mental disorders in the postpartum period
2. In comparison to routine therapy, PIP will enhance parental reflective functioning in mothers with mental disorders in the postpartum period
3. In comparison to routine therapy, PIP will enhance the rate of securely attached mother-child-dyads at 12 months’ follow-up

Subgroup analyses will be made between PIP and CAU depending on the setting (inpatient compared to outpatient/home visits). In addition, health economic analyses will be conducted for all groups (PIP as well as CAU). Therefore, the use of health and care services will be assessed, and associated costs will be estimated in order to systematically evaluate the economic burden of mothers with mental disorders in the postpartum period for the first time in Germany. Afterwards these cost results will be compared between the groups and will be related to the effectiveness results in order to get first hints on the interventions cost-effectiveness.

### Trial design

The study will be designed as a two-arm, open, multi-center RCT with parallel groups and blinded endpoint assessment (PROBE design; prospective, randomized, open, blinded endpoint) with 6 weeks of intervention.

**Fig. 1:**
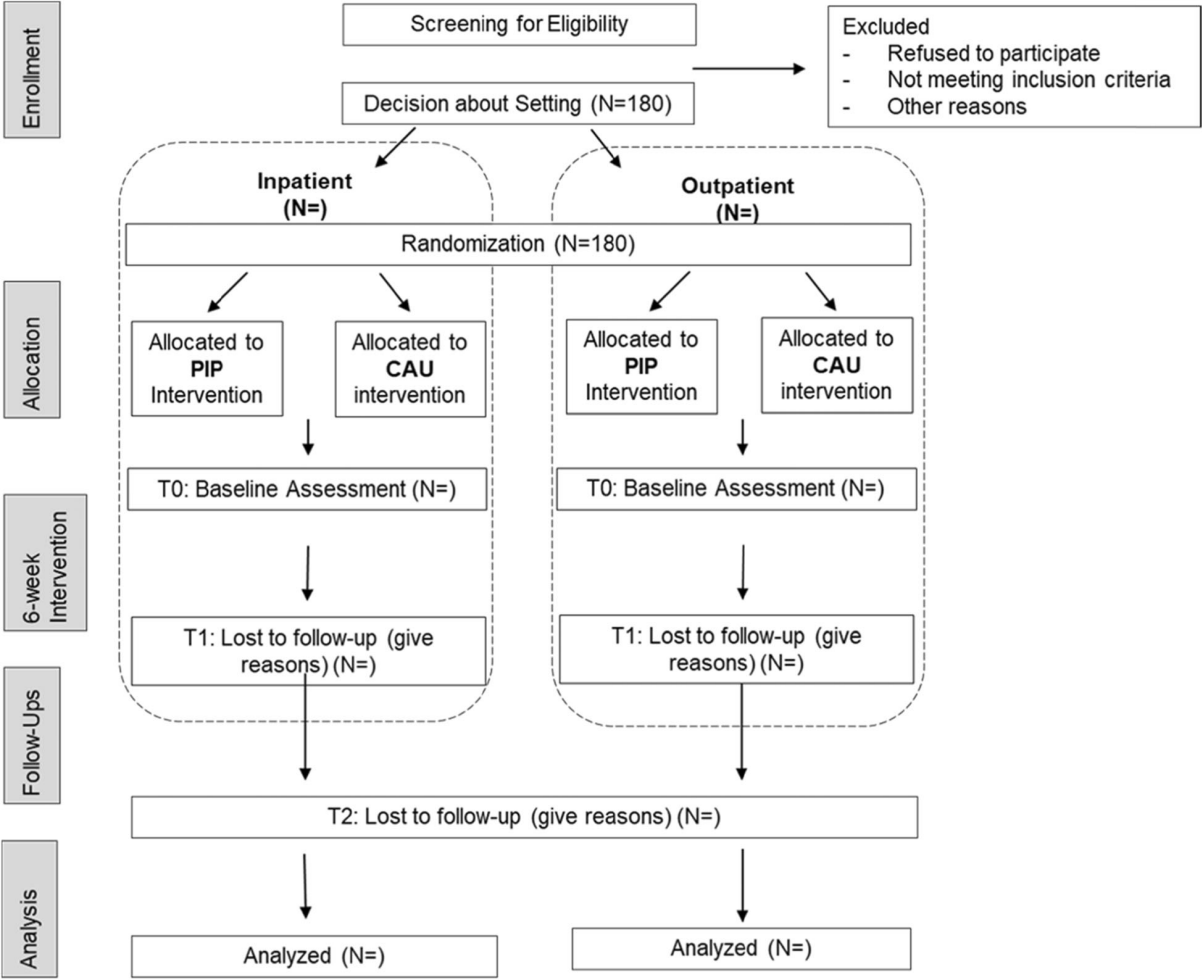
Consolidated Standards of Reporting Trials (CONSORT) diagram describing the flow of the mother-child-dyads through the study. *PIP* Parent-Infant-Psychotherapy, *CAU* care as usual

Depending on the severity of the psychopathological symptoms and in agreement with the participants’ preferences, the decision for a particular treatment setting (inpatient or outpatient) will be made before randomization. After assignment to treatment setting, participants will be randomly allocated to either the PIP intervention group or the CAU control group. A biometrician will independently carry out a 1:1 block randomization with variable block length and stratification by setting (inpatient or outpatient) and study center. Treatment allocation is masked to the assigning personnel and there will be no special criteria for discontinuing or modifying the allocated intervention. Data of mothers and children will be assessed at three measurement points (see Fig. 1): at baseline after randomization and immediately before the intervention (T0), after the intervention at 6 weeks (T1) and at 12-month follow-ups (T2). Study centers are located in the German cities Berlin and Flensburg. At the time of writing additional effort is being made for a study center in Hamburg.

### Participants

**Fig. 2:**
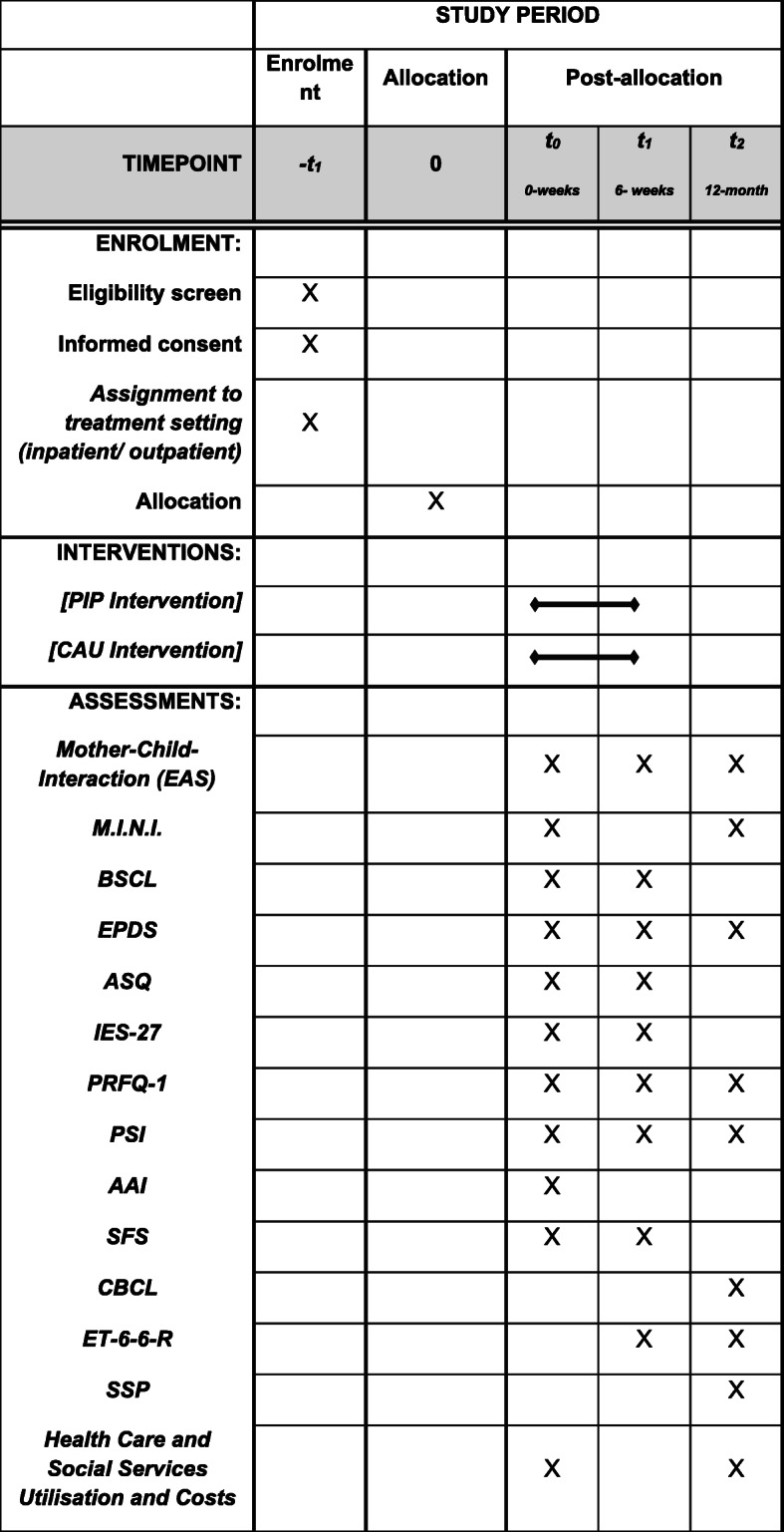
Full Standard Protocol Items: Recommendations for Interventional Trials (SPIRIT) Figure. *EAS* Emotional Availability Scale, *M.I.N.I*. Mini International Neuropsychiatric Interview, *BSCL* Brief-Symptom-Checklist, *EPDS* Edinburgh Postnatal Depression Scale, *ASQ* Anxiety Screening Questionnaire, *IES-27* Scale for impulsive behavior and emotional dysregulation of Borderline Personality, *PRFQ*-1 Parental Reflective Functioning Questionnaire, *PSI* Parenting Stress Index, *AAI* Adult Attachment Interview, *SFS* German questionnaire Crying, Feeding, Sleeping, *CBCL* Child-Behavior-Checklist, *ET6–6-R* German development test, *SSP* Strange Situation Procedure

A sample of 180 mother-child-dyads is planned to participate in the study. After screening for eligibility, written informed consent and the decision about treatment setting, the participants will be randomly allocated to either the PIP intervention arm (planned *n* = 90) or the CAU control arm (planned *n* = 90; see Fig. 2). Mother-child-dyads will receive monetary compensation of €100 to take part in the study until the 12-month follow-up. To reduce attrition, trial participants will be thoroughly informed about study goals and assessment processes. During the time of participation, the study nurse will maintain contact with the participants and schedule follow-ups. Communication (e.g., for follow-up assessments) will either be conducted by e-mail or by telephone.

Inclusion criteria will be that the participants will be German-speaking mothers with an *International Classification of Diseases, version 10* (*ICD-10)* diagnosis of a mental disorder in the postpartum period and their infants under 1 year of age. Clinical screening for eligibility and diagnoses will be made by certified therapists, physicians or psychologists at the clinical study centers before inclusion in the study. Mothers can be referred either by health services, pediatricians, clinicians or other services, or by themselves.

Exclusion criteria will be a maternal *ICD-10* diagnosis of schizophrenia, substance abuse or recent suicidal ideation. Infants will be excluded when they show symptoms of alcohol embryopathy or severe chronic organic disease. Mothers and infants will be excluded when they are participants in further clinical trials or are undergoing another form of psychotherapy.

Participants will be recruited predominantly via health visiting services and mental health services, through links with community services, and advertisements in midwife practices, and pediatricians.

### Intervention

*Focused Parent-Infant-Psychotherapy* is a psychotherapeutic intervention in which mother-child-dyads are treated together. Individual sessions could also be held together with the father. PIP aims at fostering the parent-infant-relationship by supporting the parents’ ability to understand and mentalize the child’s affective states. It aims at promoting parental self-reflection and sensitivity to support the building and maintaining of parent-infant-relationships. PIP interventions are exclusively delivered by trained and supervised psychotherapists who are additionally certified in the PIP method. The manualized PIP (Schlensog-Schuster F, Koch G, Ludwig-Körner C: SKKIPPI-Studymanual for Parent-Infant-Toddler-Psychotherapy (PIP), unpublished) is a focused psychodynamic intervention with 12 succeeding sessions of approx. 50 min in 6 weeks (two sessions per week). The aims of PIP are clarification of problems, affect regulation, self-reflection, a change of perspective and the activation of the participants’ interpersonal resources. The intervention includes a mentalization-based approach, and works with reframing, an appropriate confrontation within a supportive framework, psychoeducation, the development of action strategies, and video-feedback. To enhance manual adherence, all study therapists will be additionally trained in the manual before the start of the treatment.

The inpatient treatment is characterized by interventions in adult psychiatric departments with MBUs or day hospitals. In inpatient settings, PIP will be provided in addition to standard therapeutic care. In outpatient settings PIP will be provided in the patients’ home environment or in psychiatric outpatient departments.

### Control

Participants in the CAU group will benefit from the standard (social) pedagogical and therapeutic care provided by the German health system over the intervention period of 6 weeks. Due to ethical considerations, no “waiting-list” group will be implemented. CAU, therefore, will resample a heterogeneous class of standard interventions. As a result, more intense and therapeutic care might be provided for mother-child-dyads in the inpatient settings compared to standard care in the outpatient setting. CAU in the inpatient setting could include counseling, body or occupational therapy and other psychiatric-therapeutic interventions for the participant. The frequency of these interventions will be generally lower than PIP treatment. CAU in outpatient setting could be other therapeutic interventions or (social) pedagogical care such as developmental psychological counseling or physiotherapy. Because this might result in heterogeneities in the number and intensity of CAU, all individual interventions will be documented.

### Assessment and outcome measures

#### Primary endpoint

*Maternal sensitivity*: the primary outcome of the current study will be maternal sensitivity at the end of 6-week intervention (T1) measured by the Emotional Availability Scale (EAS). EAS [36] is a widely used assessment for the quality of mother-child interaction with excellent psychometric properties. Fifteen-minute videotaped dyadic free-play interactions will be assessed at the mother’s home environment and will be coded by independent and reliable coders blind to treatment allocation. EAS will be assessed at all three measurement time-points and will be coded using a Likert Scale (1 to 7) on maternal sensitivity as well as three further maternal dimensions (structuring, non-intrusiveness and non-hostility) and two child dimensions (responsiveness to parent and involvement with parent).

#### Secondary endpoints

*Child attachment*: the most important secondary outcome will be child attachment measured by the Strange Situation Procedure (SSP) [37]. SSP is a standardized procedure of eight episodes to record the quality of the child’s attachment. The aim of SSP is to activate the child’s attachment system. The test will be conducted in the laboratory, recorded on video, and will be coded by reliable and independent coders blind to treatment allocation. This will result in the classification of the quality of infants’ attachment behavior as being secure (B), avoidant (A) or ambivalent (C). In addition, coders are also trained to classify disorganized (D) attachment qualities. Because SSP can only be validly assessed starting at an age of 11 months, it will only be measured at the 12-month follow-up time-point. Group differences in child attachment will be examined using binarized SSP classification (secure/ insecure).

Because no cut-off values are available, dichotomous response variables for the EAS sensitivity scale at T1 and T2 will be computed using the Reliable Change Index (RCI) as additional secondary endpoints (RCI > 1.96, see [38]).

Further secondary outcomes for the maternal psychopathological symptoms:

- *M.I.N.I.* – The Mini International Neuropsychiatric Interview (M.I.N.I.) [39] is a structured diagnostic interview which assesses 20 psychiatric disorders by the *Diagnostic and Statistical Manual of Mental Disorders, 4th edition* (*DSM-IV)* and *ICD-10* diagnosis. The M.I.N.I. is used to verify the clinical diagnoses and will be administered at the mother’s home environment by trained interviewers at T0 and T2
- *BSCL* – The Brief-Symptom-Checklist (BSCL) [40] is a validated questionnaire for assessing maternal psychopathological symptoms. The BSCL will be measured at T0 and T1
- *EPDS* – The Edinburgh Postnatal Depression Scale (EPDS) [41] is a standard screening instrument to measure maternal symptoms of postnatal depression. The EPDS will be assessed at all three measurement time-points. Dichotomous response variables will be computed according to the cut-offs reported in [41] (EPDS < 12)
- *ASQ* – Maternal symptoms of anxiety disorder will be measured by the Anxiety Screening Questionnaire (ASQ) [42]. The ASQ is a questionnaire which assesses all anxiety disorders diagnosed by *DSM-IV* and *ICD-10.* It will be measured at T0 and T1
- *IES-27* – For assessing Borderline Personality Disorder, the Scale for impulsive behavior and emotional dysregulation of Borderline Personality Disorder (IES-27) [43] is used. The IES is a screening instrument which measures typical behavioral and perceptual patterns of this disorder. The questionnaire will be assessed at T0 and T1
- *PRFQ-1* – The Parental Reflective Functioning Questionnaire (PRFQ) [44] is a validated questionnaire which measures the mother’s ability to mentalize in context of the relationship to her child. The questionnaire will be assessed at all three measure times. Because no cut-off values are available, dichotomous response variables for the PRFQ will be computed based on the RCI > 1.96 criterion [38]
- *PSI* – The German version of the Parenting Stress Index (PSI) [45, 46] is a questionnaire which measures the maternal stress at T0, T1 and T2. Because no cut-off values are available, dichotomous response variables for the PSI will be computed based on the RCI > 1.96 criterion [38]
- *AAI* – Maternal attachment style and reflective functioning, i.e., the capacity to mentalize, are considered as important moderators in the present design and are assessed using the Adult Attachment Interview (AAI) [37]. AAI is a semi-structured interview with excellent psychometric properties to evaluate the maternal “state of mind” with respect to attachment [37]. AAI will be conducted in the laboratory by trained interviewers at baseline (T0), transcribed, and subsequently scored by reliable and independent coders blind to treatment allocation

Secondary outcome measures for the child:

- *SFS* – The infant’s symptoms are measured by using the German questionnaire Crying, Feeding, Sleeping (SFS) [47]. The SFS assesses problems in the child’s behavioral regulation and dysfunctional communication patterns, and stress symptoms of the primary caregivers. The questionnaire will be assessed at T0 and T1
- *CBCL* – To detect the child’s emotional and behavioral problems, the Child-Behavior-Checklist (CBCL) [48] is used. The CBCL is validated at a child’s age of 18 months and will, therefore, be assessed at 12-month follow-up only
- *ET-6-6-R* – The level of social-emotional, cognitive, motor and language development of the child is assessed by the German development test ET6–6-R [49]. The ET-6-6-R is a standardized test for children aged from 6 months to 6 years that measures the developmental status of the child. The test will be measured at the mother’s home environment at T1 and T2.

#### Further secondary outcomes

*Treatment adherence* will be measured using an in-house-developed, short, 30-item Treatment Adherence Questionnaire. PIP therapists are asked to evaluate after each session the methods and interventions applied

*Health care and social services utilization and costs*. To estimate the economic consequences on the health care service usage in Germany, a cost analysis will be carried out in both groups by using a self-developed resource consumption questionnaire. Questions about the utilization of health and social care and demand on early intervention services are assessed. The questionnaire will be applied at T0 and T2. These data are used for calculation of health care costs. Therefore, these resources will be monetarily assessed by using standardized unit costs from a societal perspective, which will be calculated based on regularly published sources.

### Samples size calculations and statistical analyses

A power analysis [G*Power] based on a reported moderate to large effect size of Cohen’s *d* = 0.64 for a difference in maternal sensitivity after 6 weeks of intervention between the intervention group and the CAU group [19] and two-tailed testing with a significance level of *α* = 5% using an analysis of covariance (ANCOVA) (with maternal sensitivity at T1 as the dependent variable and maternal sensitivity at T0 as a covariate) resulted in a required sample size of *n* = 70 (per group) to detect an effect with a power of 95%. With an assumed drop-out rate of about 20% [1] the sample size calculates to *n* = 90 per group (total sample size *n* = 180 mother-child-dyads).

The statistical analysis will be carried out at the end of the last follow-up assessment and all analyses will be conducted in accordance *with intention-to-treat (ITT)* principles and will be including all randomized mother-child-dyads in the study. The *Full Analysis Set (FAS) will be used for all analyses (unless otherwise stated),* it will include all randomized mother-child-dyads. Data will be analyzed according to randomization irrespectively of the treatment received. Data from mother-child-dyads that have withdrawn from the study will be included in the FAS up to the date of their termination. The *Per-Protocol (PP) population* will comprise a subset of the FAS. It will consist of mother-child-dyads who will have received treatment according to the randomization, will have adequate therapy adherence, and no major protocol violations. Major protocol violations will be considered as not meeting all inclusion criteria, a change of treatment group or as the PIP intervention that has not been carried out properly according to the treatment manual. The *safety population* will include mother-child-dyads who have participated in at least one intervention session. Participants in the safety population will be analyzed according to the treatment actually received.

#### Primary analysis of the primary outcome

The evaluation of the primary outcome (EAS, maternal sensitivity at T1) will be performed by a covariance analysis (ANCOVA) with the independent variable treatment (PIP vs. CAU), adjusted for maternal sensitivity at baseline (T0) and treatment setting as stratification variable based on the FAS population. Adjusted mean values of treatment groups with 95% confidence intervals and *p* value (two-sided) for group comparisons will be presented.

#### Secondary analyses of the primary outcome

The primary analysis will be repeated as described with the PP population. In addition, the effect of treatment setting on the efficacy of PIP will be investigated by means of a moderator analysis. Moderators show their effect in the interaction with treatment group. Thus, the model will include all variables as described for the primary analysis with the addition of the treatment*setting interaction. Additional moderator analyses regarding the effectiveness of PIP including maternal attachment style and reflective functioning (AAI) as moderators will be calculated. A sensitivity analysis of the primary outcome will be computed using multiple-imputation methods and the PP population adjusted for maternal sensitivity at baseline and treatment setting as stratification variable. In a further sensitivity analysis of the primary outcome, a hierarchical regression model will be calculated which, in addition to the adjustment for the baseline values and the treatment setting, will include the therapists who performed the intervention in the model. Both sensitivity analyses will be repeated using also study center as stratification variable.

Linear ,mixed models for longitudinal data will be used to examine the change of the primary outcome across all measurement time-points for the treatment groups. The model will include predictors for treatment group and time as fixed effects as well as the treatment group × time interaction, baseline maternal sensitivity as covariate, treatment setting as stratification variable, and subjects as random effects. The therapists and further predictors will be added to the model when indicated by goodness-of-fit model comparisons (based on Akaike Information Criterion (AIC) values). All secondary analyses of the primary and secondary outcomes are considered exploratory.

#### Analyses of the secondary outcomes

Secondary outcomes will be computed according to the analysis strategy of the primary outcome using ANCOVA or logistic regression (depending on the scale of the respective outcome, continuous or dichotomous). Likely moderators will be added to the models including setting, and maternal attachment style and AAI reflective functioning. In order to investigate the longitudinal course until the 12-month follow-up, linear mixed models will also be calculated for the change of secondary outcomes (e.g., EPDS, PSI and PRFQ). In addition, sensitivity analyses are planned for the health economic analyses, in which the underlying unit-cost assumptions will be varied within realistic ranges.

#### Missing data

Analyses of primary and secondary endpoints will be conducted based on the FAS population without imputation of missing data. A sensitivity analysis is computed by imputing missing data of the primary outcome with multiple-imputation methods for linear regression using Markov chain Monte Carlo (MCMC) simulations. The selection of imputation variables will be decided blinded to treatment allocation and at least 5000 iteration and 100 imputations will be used.

### Monitoring and trial management

A monitoring of the study centers will be performed based on the case numbers and data collection by the study teams (mutual monitoring of the study centers) consisting of the local principal investigator (PI) and the local researchers (the local Trial Management Group, TMG). The TMGs are responsible for the identification, recruitment and follow-up of study participants, data collection and adherence to the study protocol and will meet weekly to review trial conduct. The results of the monitoring are an important aspect of the bi-annual consortium meetings of the SKKIPPI project including all leading PIs, the biometrician, the data manager and the researchers. An independent external advisory board, consisting of three international experts, will meet annually to audit and monitor the project process. While the TMG, Advisory Board and SKKIPPI consortium operate independent of the sponsor, an additional external monitoring is also carried out by the sponsor based on case numbers and reached milestones.

Data quality will be continuously monitored and the study supervised by the Institute for Social Medicine, Epidemiology and Health Economics of the Charité – Universitätsmedizin Berlin (SEGC). The SEGC Monitoring Group consists of the independent biometrician (SR) and the data manager who will keep the researchers and outcome coders blinded for treatment allocation and will monitor data safety. Outcome data will be unblinded only after the last participant has finished the study and no interim analyses of the primary and secondary outcomes are planned. Only pseudonymous data sets will be analyzed.

There are formal stopping rules for the trial in case that a severe adverse event (SAE) occurs. In case of a SAE the Advisory Board will be consulted. The consortium leader (LK) will report any SAE to the Local Ethics Committees and all relevant parties. Standard operation procedures (SOPs) will be activated and will lead to exclusion of the participant and potentially to the termination of the trial.

### Methods to minimize bias

Data will be stored electronically at SEGC and will remain blind for treatment allocation for all researchers and coders. The database of participant information and allocation will be maintained by the SKKIPPI study nurse at the study center in Berlin. The study nurse will be the only person who will have access to the participants’ personal data and the treatment allocation list. To minimize research bias only trained psychologists will notify mothers about the allocation outcome. Data will be collected by trained researchers who will be blind for randomization and treatment allocation and who will not be involved in the treatment of the mother-child-dyad.

The study sets a high standard in PIP treatment and minimizes therapeutic bias and attrition insofar that all study therapists will be trained in the manual before the beginning of treatment and will take part in monthly supervision by experienced PIP therapists. For monitoring the therapeutic process, all therapists will complete the Treatment Adherence Questionnaire after each treatment session. In order to create a good therapeutic alliance, therapists will provide a comfortable and trustful atmosphere. It will be ensured that the patient is aware of their therapeutic progress and own expectations. To minimize bias for the assessments, most of the data will be assessed electronically and stored in a secured database supervised by the SEGC. In addition, the study has a strict separation of research assessments and therapeutic intervention and researchers are well-trained in the assessment methods.

### Ethical considerations and limitations

Data will be assessed, processed and stored according to the data protection laws and the local data protection protocol. The research project will be carried out in compliance with the study protocol and the Declaration of Helsinki (1996 version, Somerset West). All researchers and participants in the study are obliged to follow the rules of Good Clinical Practice (ICH-GCP). Participants will only be enrolled in the study after giving inform consent. The study is designed as an open RCT with blinded endpoint assessment and blinded analysis. Still, participants will be aware of their treatment group allocation.

This trial compares a psychotherapeutic intervention with available CAU interventions. The CAU interventions will be heterogeneous and administered under very diverse conditions. Therefore, it will be difficult to document which component of CAU is most effective. Moreover, there will be differences in CAU depending on the setting (inpatient vs. outpatient). Due to ethical concerns participants will not be randomly allocated to a setting. Thus, more severe cases will be evident in the inpatient setting, lowering the representativeness of the results. At the same time the CAU routine intervention will be more intense in inpatient departments, i.e., CAU in inpatient settings will likely include other (psycho-)therapeutic interventions. Furthermore, ethical consideration precluded the inclusion of a waiting-list group. This very same concerns will also lead to the fact that all mother-child-dyads allocated to CAU will be offered an intervention or CAU. Thus, the standard in the CAU will likely be higher than in the overall population in Germany which will bias generalizability of the results. In order to minimize these effects and to incorporate these limitations in the analyses, all interventions in all conditions will be documented.

There is a lack of good quality trials for the proof of efficacy of PIP in the treatment of mothers [15] but more specifically of fathers [24]. This present study is designed for mothers with postpartum mental disorders. The importance of the fathers’ involvement for treatment outcomes, for the family system and the impact on child’s development is not assessed, neither will father-infant-dyads be included in the study. Following the study by Letourneau [50, 51] mothers with PPD report that fathers are a key resource for support. Until now, little is known about the fathers’ effects on the system, and with the present design no conclusions regarding fathers can be drawn.

A further limitation of this study is the eligibility of a diverse range of maternal postpartum diagnoses which will likely lower the power to detect effects. On the other hand, this design was chosen because of the higher generalizability when not restricting inclusion criteria to single diagnoses. If sufficient data are available, exploratory analyses of subsamples with the same diagnoses are intended. The PIP intervention in the present study has been designed as a short-term treatment of 12 sessions for parent-infant-dyads. Short, sensitivity-based interventions have been shown to have superior efficacy compared with longer ones [15, 19]. Still, in the case of a highly traumatized mother it might be less effective. Of note is also that study therapists and participants will not be blind to treatment allocation.

Some studies [23, 52–54] point out that there is a link between the engagement of participant, withdrawal and therapeutic alliance. In this trial, therapeutic alliance and patients’ motivation will not be assessed. But all withdrawals and severe events will be documented and contribute to the statistical analyses.

## Discussion

Interventions with a focus on attachment and maternal sensitivity and, in particular, parent-infant-psychotherapeutic interventions, have a good evidence base for the treatment of mental health problems in the postpartum period [15, 19] and the foundation of healthy child development. Given the high prevalence of postpartum mental disorders in mothers and the adverse impact on the child, a high-quality study focusing on the efficacy of PIP in Germany is needed. PIP in inpatient psychiatric departments has not been in the focus of controlled trials so far, and the present study differs to previous ones in that no particular risk samples are examined (like social adverse samples [1] or imprisoned mothers [25]) but diagnosed mothers with mental disorders in the postpartum period. There is a strong focus on prevention studies in early childhood in the German health care services. For the particular case of mental disorders, the trial is expected to reveal the efficacy of PIP in comparison with CAU in inpatient psychiatric settings and also in outpatient home visits to contribute to the ongoing discussion in the German health care system. As a further result, it will be expected to provide information for practitioners, and mothers and fathers on factors contributing to the efficacy of PIP interventions and to improve the future care situation for patients and their families in Germany.

### Trial status

The recruitment for the trial started in January 2019 and is ongoing (as of May 2020). It is expected to be completed in September 2021. This protocol is version 1.0, dated 13 September 2018.

## Data Availability

Trial results will be published in peer-reviewed journals regardless of outcome and will be presented at national and international conferences. An accessible summary of the findings will be published for participants, and members of the public at the project website skkippi.de. The data sets analyzed in the current study will be made available from the corresponding author on reasonable request in anonymized format only. A model consent form and other related documentation given to participants and authorized surrogates will be available from the corresponding author on reasonable request.

## Abbreviations

SKKIPPI: Parent-Infant-Psychotherapy in cohort- and intervention studies [Eltern-Säugling-KleinKInd-Psychotherapie mittels Prävalenz- und Interventionsstudien]
PIP: Parent-Infant-Psychotherapy
RCT: Randomized controlled intervention trial
CAU: Care as usual
PPD: Postpartum depression
MBU: Mother and Baby Unit
AAI: Adult Attachment Interview
PRFQ: Parental Reflective Functioning Questionnaire
SSP: Strange Situation Procedure
PROBE design: Prospective, randomized, open, blinded endpoint
EAS: Emotional Availability Scale
M.I.N.I.: Mini International Neuropsychiatric Interview
BSCL: Brief-Symptom-Checklist
EPDS: Edinburgh Postnatal Depression Scale
ASQ: Anxiety Screening Questionnaire
IES-27: Scale for impulsive behavior and emotional dysregulation of Borderline Personality
PSI: Parenting Stress Index
SFS: German questionnaire Crying, Feeding, Sleeping [Schreien, Füttern, Schlafen]
CBCL: Child-Behavior-Checklist
ET6–6-R: German development test
ANCOVA: Analysis of covariance
ITT: Intention-to-treat
FAS: Full Analysis Set
PP: Per-protocol
RCI: Reliable Change Index
AIC: Akaike Information Criterion
MCMC: Markov chain Monte Carlo
PI: Principal investigator
TMG: Trial Management Group
SAE: Severe adverse event
SOP: Standard operation procedure
SEGC: Institute for Social Medicine, Epidemiology and Health Economics of the Charité
ICH-GCP: Rules of Good Clinical Practice
DRKS: German Register for Clinical Trials [Deutsches Register Klinischer Studien]
eCRF: Electronic Case Report Form
